# Using Color, Texture and Object-Based Image Analysis of Multi-Temporal Camera Data to Monitor Soil Aggregate Breakdown

**DOI:** 10.3390/s17061241

**Published:** 2017-05-30

**Authors:** Irena Ymeti, Harald van der Werff, Dhruba Pikha Shrestha, Victor G. Jetten, Caroline Lievens, Freek van der Meer

**Affiliations:** Department of Earth Systems Analysis, Faculty of Geo-Information Science and Earth Observation, University of Twente, P.O. Box 217, 7500 AE Enschede, The Netherlands; harald.vanderwerff@utwente.nl (H.v.d.W.); d.b.p.shrestha@utwente.nl (D.P.S.); v.g.jetten@utwente.nl (V.G.J.); c.lievens@utwente.nl (C.L.); f.d.vandermeer@utwente.nl (F.v.d.M.)

**Keywords:** soil aggregate, Huang technique, entropy, multi-temporal, proximal sensing, freezing–thawing, rainfall

## Abstract

Remote sensing has shown its potential to assess soil properties and is a fast and non-destructive method for monitoring soil surface changes. In this paper, we monitor soil aggregate breakdown under natural conditions. From November 2014 to February 2015, images and weather data were collected on a daily basis from five soils susceptible to detachment (Silty Loam with various organic matter content, Loam and Sandy Loam). Three techniques that vary in image processing complexity and user interaction were tested for the ability of monitoring aggregate breakdown. Considering that the soil surface roughness causes shadow cast, the blue/red band ratio is utilized to observe the soil aggregate changes. Dealing with images with high spatial resolution, image texture entropy, which reflects the process of soil aggregate breakdown, is used. In addition, the Huang thresholding technique, which allows estimation of the image area occupied by soil aggregate, is performed. Our results show that all three techniques indicate soil aggregate breakdown over time. The shadow ratio shows a gradual change over time with no details related to weather conditions. Both the entropy and the Huang thresholding technique show variations of soil aggregate breakdown responding to weather conditions. Using data obtained with a regular camera, we found that freezing–thawing cycles are the cause of soil aggregate breakdown.

## 1. Introduction

Soil aggregate breakdown is a function of soil strength and the kinetic energy of the rainfall [1,2]. Soil strength depends on soil particle distribution, structure, soil organic carbon, ionic bridging, clay and carbonates. Different land cover or land use, such as forest or agriculture, influences soil structure and organic matter content [3]. Other properties may relate to climate (e.g., high calcium carbonate content in drier environment) or soil processes (e.g., high iron oxide content in Ultisols or Oxisols). Aggregate stability also decreases when water dispersible clay content increases in relation to total clay, as reported in [4]. Weather conditions influence soil aggregate, especially when both rainfall and freezing–thawing cycles occur [5]. While rainfall induces soil aggregate breakdown, compaction and crusting, freezing–thawing cycles particularly affect formation and destruction of soil structure [6,7]. During a rainfall event, soil macro-aggregate (>250 μm) breaks down into smaller aggregate [8,9,10].

Rainfall can destroy soil aggregate via two processes: (i) the direct impacts of water drops mechanically disintegrate aggregate into smaller particles leading to surface sealing; and (ii) the small aggregates are submerged rapidly during a rainfall, air is trapped and aggregates implode [11]. While a structural crust is formed when aggregates are reorganized with limited particle displacement, a sedimentary crust results from clay particle displacement [12]. Both mechanisms help to create a thin crust layer with a light color, high bulk density and low porosity [13,14,15]. In fact, soil detachment by raindrops is a start of interrill erosion and surface seal formation. Detecting and monitoring soil aggregate breakdown requires a micro-plot scale having an area of some hundreds of square centimeters. It is already known that ground-based methods are time-consuming and expensive.

Remote Sensing (RS) has shown potential to assess several soil properties such as moisture, organic matter, iron, sand and clay [16,17,18,19,20]. Microwave RS has been used to determine soil moisture and soil surface roughness [21,22,23]. To represent the soil surface roughness as standard deviation of elevation data, optical photogrammetry is utilized [24,25,26]. There are RS studies that have investigated the effect of natural rainfall on surface roughness. Calculating surface roughness indices such as standard deviation of height (vertical component) and autocorrelation length (horizontal component of roughness spectra) from Digital Surface Models (DSM) obtained from a plowed and harrowed field, Marzahn et al. [27] reported a decline in roughness due to rainfall. Recently, the Structure from Motion (SfM) approach is used to calculate the roughness parameters on a 2 × 3.4 m^2^ plot over three months based on the Fourier transform of the digital elevation model (DEM). This research concluded that roughness changes, due to weather conditions, happened at spatial resolution below 50 cm [28]. To quantify changes in soil surface structure associated with macro-aggregate breakdown, Croft et al. [29] combined laser scanner and hyperspectral data measured at a variety of viewing and illumination solar angles. They found that, due to crusting, the surface roughness declines and the effect of shadow-casting by soil aggregate reduces. In addition, Moreno et al. [30] concluded that shadow analysis is a suitable technique to assess the soil surface roughness decline of a sandy clay loam soil after tillage operation.

Using RS approach changes on soil aggregate breakdown occurring over a short period of time can be detected. To understand these changes, it is important to monitor the interaction between soil surface and the surrounding environment at high temporal resolution. In addition, considering that data acquisition still remains expensive and image analysis are often complicated and time-consuming, we investigated the possibility to monitor soil aggregate breakdown in a straightforward and cost-effective way. To achieve our goal, we used a red, green, blue (RGB) Single-lens reflex (SLR) camera mounted in a fixed setup to enable photographing the same location over time acquiring time series data acquisition. Next, we examined the capability of the RGB SLR camera to monitor soil aggregate breakdown in soils of different texture classes under natural conditions at a micro-plot scale at daily basis.

## 2. Materials and Methods

### 2.1. Experimental Setup

To monitor aggregate breakdown under natural conditions, we designed an outdoor experiment consisted of a RGB SLR camera and a weather station both mounted on a tripod. In addition, below the tripod five trays filled with soil were placed. The soil samples were collected in the end of September 2014 in Limburg (Silty Loam with various amount of organic matter content (OM) and Loam) and Deventer (Sandy Loam), both in The Netherlands. Soil aggregate stability was performed on soils according to ISO 10930 [31], to provide a realistic analysis of the structural stability of soil aggregates when subjected to the action of weather, cultivation, etc. and to enable the soils to be classified on the basis of the stability of their aggregates. The results obtained showed that the Soil 1 (Silty Loam) was very unstable while the rest of the soils were unstable. In order to see the effect of land use/land cover on soil aggregate stability samples were collected from topsoil (20 cm) of agricultural fields as well as forest area. The soil particle size fraction determination was carried out on fine earth (<2 mm) according to ISRIC protocol [32]. The OM was oxidized with H_2_O_2_. Sand was separated from clay and silt with a 50 μm sieve. The clay and silt fractions were determined with pipette method based on sampling a 1 liter suspension with a 20 mL pipette. The obtained clay, silt and sand fraction are calculated on a dry-ash-free basis. The OM was determined by heating the sample at 600 °C for more than 12 h and calculating the weight loss on the dry soil. Table 1 summarizes soil characteristics used in this study.

**Scheme 1:**
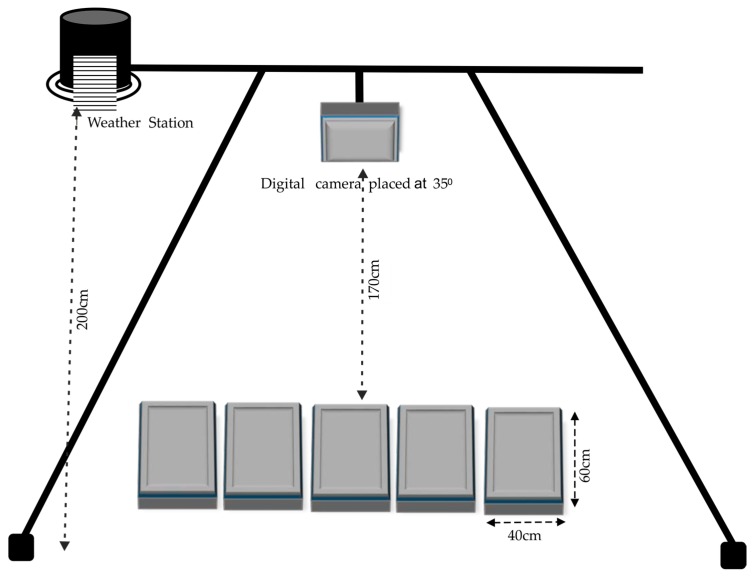
Schematic design of the outdoor experimental setup. On the tripod are both the weather station (on the left) and the camera (in the center) placed at an angle of 35°. Next to the tripod are the undisturbed soil trays photographed each day. From left to right, the Silty Loam with low OM content, Silty Loam with high OM content, Loam and Sandy Loam are placed.

The soil samples were air-dried at room condition for four weeks. Five trays of 60 × 40 × 5.5 cm^3^ were filled manually with soil using a small shovel. Because of this procedure, soil aggregates of various sizes were randomly distributed. When placed at the outdoor experimental site, the trays were tilted at an angle of approximately 10 degrees to allow the trays to drain. Drainage was enabled by 5 mm diameter holes drilled in one side of the tray at the bottom. To avoid soil leaking out, panty hose filters were used. Together with the camera, a DaVIS Instruments ISO 9001 [33] weather station was installed 2 m above ground to record rainfall and air temperature data at a thirty minutes interval. Five trays (60 × 40 × 5.5 cm^3^) filled with soil of different texture and OM content were photographed each day at 15:00, as shown in Scheme 1. These bare soils were kept undisturbed for photographing. We collected images from November 2014 to February 2015.

### 2.2. Image Acquisition

**Figure 1:**
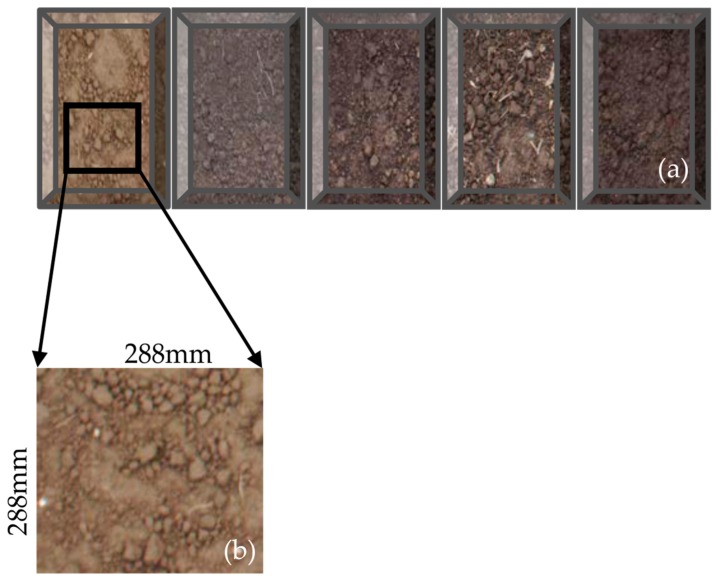
Example of an image selected for analysis: the soil trays photographed on 15 November 2014 (**a**); and one of the five 288 × 288 mm^2^ image subset with a pixel size 1.8 mm (**b**).

Since our goal was to monitor soil aggregate breakdown, a Canon EOS 600 time-lapse camera housing was mounted on a tripod above the trays. The camera had a 60° and 43° horizontal and vertical field of view, respectively. To avoid influencing the rain, the camera was placed with an angle of approximating 35°, making an oblique capture of the trays. The camera took photos each day, at 15:00 local time with a solar elevation varied between 13.6° and 17.5° (with lowest solar elevation of 7.5° on 16 December) while the azimuth changed from 220.6° to 213.9° on 4 November 2014 and on 10 February 2015, respectively. Calibration panels were present in the field of view. The panels however often were saturated in the image and also appeared to darken with moist. The camera had an 14-bit A/D converter delivering 8-bit (RGB) data. With this dynamic range, there was not a single set of camera settings that could answer to all illumination conditions. A histogram-matching of the data was hence performed when needed in the subsequent time-series analysis (see below in Section 2.5). Given the approximated three months duration of the experiment, the solar elevation and azimuth change was 3.9° and 6.7°, respectively. A further correction for this change, as well as BRDF differences between the trays, was therefore not implemented. The images were subset to the middle of the tray to avoid seeing the edge, as shown in Figure 1. ImageJ software provides a tool which convert an image from pixels to any metric units. Knowing the distance in pixel and the real distance (for e.g., in millimeters) of the image, the spatial resolution of this image can be calculated. The images of 512 × 512 pixels correspond to a 288 × 288 mm^2^ area with a spatial resolution of 1.8 mm/pixels.

**Figure 2:**
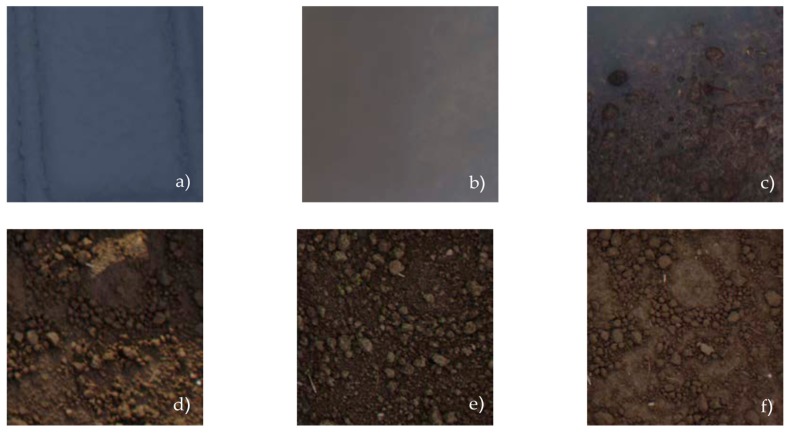
Examples of images that are discarded: snow (**a**); fog (**b**); standing water (**c**); sunlight distribution (**d**); and frozen surface (**e**). The last image (**f**) is an example of an image accepted for further analysis.

Images that could not be used for analysis because of snow cover, fog or standing water, were discarded after visual inspection. Additionally, the basic statistics such as minimum, maximum, mean and standard deviation were calculated for each image. These statistics were performed considering the three bands (red, green and blue). Images influenced by direct sunlight or a frozen soil surface had higher standard deviation (digital number) value compared to an accepted image for further analysis (Figure 2f). Therefore, the images with a standard deviation higher than 15 for Soil 1 and Soil 4, 10 for Soil 2 and Soil 5 and 13 for Soil 3 were discarded (Figure 2). These thresholds were derived experimentally.

### 2.3. Shadow Ratio

Various soil properties affect soil’s spectral reflectance such as soil particle distribution, organic matter content, soil moisture, iron oxide, soil minerals, presence of salts and soil crusting. The influence of soil structure on soil reflectance has been investigated on sieved soil under controlled laboratory condition. Banninger et al. [34] and Wu et al. [35] have shown that the reflectance decreases with increasing soil particle size or lump size. Soil roughness affect the soil optical properties, because more light is kept in the space between the coarse soil aggregates in comparison to finer aggregates or grains. In the field, soil aggregates of various size make the soil surface rough. Soil aggregate lead to self-shadowing of the soil surface resulting in a low reflectance value [36]. Cierniewski [37] found that the shadowing coefficient of soil surface, which is the proportion of shadowed area per unit area of soil fragments, decreased with a decrease of soil roughness. In addition, the reflectance of a rough soil surface diminishes with increasing sun angle [38,39]. However, if the soil surface is smooth, the reflectance at any sun angle is a function of the color, soil aggregate and Bidirectional Reflectance Distribution Function (BRDF). Here, no BRDF correction was performed. In the visible spectrum, light scatters more in blue band compare to green and red bands. Therefore, the blue band has relatively higher intensity value than the red band in shaded areas. Using image band ratio (blue/red), assuming that the pixels in shadowed regions have higher intensity values than those pixels in non-shadowed region, shadow cast could be detected. Considering that the soil surface roughness causes shadow cast the mean of blue/red band ratio was used to investigate changes of soil aggregate over time. This band ratio (blue/red) was performed using IDL ENVI 5.2 [40]. The shadow ratio was calculated for each pixel in an image with 512 × 512 pixels. After the image with shadow value was obtained, the mean and standard deviation of this image are used for further analysis.

### 2.4. Gray Level Co-Occurrence Matrix: Entropy

Image texture describes spatial variation in an image. The gray level co-occurrence matrix (GLCM) is used here to describe image texture [41]. The GLCM implementation in IDL ENVI 5.2 requires selection of a gray level quantization (intensity), an angle to deal with anisotropy and a window size. The matrix must contain a gray quantization level in order to obtain a statistically reliable estimate. More gray levels give more details, but higher gray level quantization also increases computation time. Therefore, a 32 gray level quantization was used in this research. The GLCM in IDL ENVI 5.2 considers multiple orientations (0°, 45°, 90°, and 135°). In our research, the angle of 135° captures the local variation between neighboring pixels. The direction of GLCM represents the occurrence of patterns. These patterns are caused by reflectance variations due to soil aggregates shadowing at 15:00. Therefore, the sun angle-target-camera position during image acquisition is important for GLCM. In addition, the direction of 135° of GLCM indicates that the soil aggregates start to break down at the edges. Dealing with various soil aggregate size, an optimal window size is required. Sometimes, the soil aggregate is larger than a chosen window size meaning that the GLCM is performed on a homogeneous area. Using the first image of each soil dataset, the window size of GLCM was determined when maximal entropy value was reached. Due to different soil aggregate size, the window size of GLCM varies depending on the soil dataset used. While the window size of 27 × 27 pixels was chosen for Soil 1, Soil 2 and Soil 3, for Soil 4 and Soil 5 the window 29 × 29 pixels and 25 × 25 pixels were selected, respectively. The GLCM calculation was performed on the mean of three bands (red, green and blue) using IDL ENVI 5.2. Once the GLCM was calculated, descriptive features such as contrast, dissimilarity, homogeneity, energy, entropy, mean, variance and correlation are derived from this matrix [42]. To monitor and characterize disintegration of weak rocks, Rincon et al. [43] reviewed various image texture features and chose entropy. This research showed that the entropy is an appropriate texture feature for assessing and quantifying the degree of disintegration of the weak rocks. This study demonstrated that color intensities of an image change due to disintegration of weak rocks. Cracks and void space lead to more light absorption and to lower entropy value. This intensity change in an image is reflected by entropy. Since our goal is similar to [43], we selected the GLCM entropy to investigate soil aggregate breakdown. The entropy is a statistical measure that quantifies the amount of uncertainty in an image and is calculated using Equation (1).

(1) $$\mathrm{Entropy}=-\;\overset{N_{G-1}}{\underset{i=0}{\sum }}\overset{N_{G-1}}{\underset{j=0}{\sum }}p\left(i,j\right)\log\left(p\left(i,j\right)\right)$$

where *G* is gray level, *p* is probability of GLCM, *i* is the intensity in the X direction of GLCM, and *j* is intensity in the Y direction of GLCM.

The maximal entropy is reached when all probabilities in a matrix are equal. Minimum entropy is achieved when the image is constant meaning all of the pixels have the same gray level. Therefore, the entropy is defined within the limits 0 ≤ Entropy ≥ log_2_*G*. The maximal entropy is not a fixed value because it depends on the *G* (gray level) used. The entropy is calculated within a window size and its value is assign to the center pixel of the window. This procedure is repeated until the pixels in an image have an entropy value. However, at the edges of the image, depending on the window size used, the calculation of entropy is not performed. After the image with entropy values was obtained, the mean and standard deviation of this image were used for further analysis. We consider soil aggregate breakdown as a random process, because it is difficult to identify how it changes under natural conditions. Being a measure of dispersion of a random variable, the GLCM entropy might reflect the process of soil aggregate breakdown. While a high entropy value shows dispersed distribution, a low entropy value indicates a compact distribution of considered phenomenon (soil aggregate breakdown). At the beginning of the experiment, the soil surface was covered with aggregates of various size randomly distributed. As a result, the image is texturally heterogeneous indicating a high entropy value. Due to weather conditions, the soil aggregate breaks down over time resulting in a smooth soil surface. Thus, the image tends to become constant meaning all the pixels have a similar gray level indicating a low entropy value.

### 2.5. Object-Based Image Analysis: Huang Thresholding

When an image has a sufficient high spatial resolution, pixels are smaller than the object so grouping of pixels is possible in order to obtain real-world homogeneous features [44]. Object-based image analysis (OBIA) approach, which allows estimation of the image area occupied by soil aggregates, was used. OBIA consider not only the spectral reflectance and neighbor relations, but also the shape and the size of objects [45,46]. Since our interest is monitoring soil aggregate breakdown, choosing the 1st image of each soil dataset as reference (start of the experiment) is a reasonable choice. In addition, images acquired during a day have significant variations caused by illumination differences or changing weather conditions. Therefore, when comparing physical changes in surface properties from different dates, it is necessary to exclude these variations. This is achieved by normalizing the images with the reference image. Such normalization was performed using histogram equalization and histogram matching [47,48,49]. First, histogram equalization was applied to the reference image. Next, the other images in the dataset were imposed to the equalized reference image using histogram matching plugins in ImageJ 1.51c [50]. After images were made comparable to each other, soil aggregates separation from background was required in order to estimate the area they occupy in an image. This was achieved by using a histogram thresholding technique, which classifies the pixels in two classes: object (soil aggregate) and background. When the histogram has no clear separation between object and background, the threshold could be determined using fuzzy theory approach [51,52]. We selected the Huang thresholding technique [53] to identify an appropriate threshold that divides soil aggregate from the background. The Huang thresholding method is based on the fuzzy theory and membership function μ_A_(x_mn_) ∈ [0, 1] where A is a fuzzy subset of an image X (M × N) and x_mn_ is a pixel with a gray level in X. The fuzzy subset A is associated with each pixel x_mn_ of the image X. The membership function for a pixel with gray level (x_mn_) is assigned by:

(2) $$\mu_{A}(X_{\mathrm{mn}})=\frac{1}{1+\left|\left(x_{\mathrm{mn}}\right)-\mu_{b}\right|/C}\;\mathrm{if}\;(X_{\mathrm{mn}})\;\leq \mathrm{threshold}$$

(3) $$\mu_{A}(X_{\mathrm{mn}})=\frac{1}{1+\left|\left(x_{\mathrm{mn}}\right)-\mu_{o}\right|/C}\;\mathrm{if}\;(X_{\mathrm{mn}})\;>\mathrm{threshold}$$

where C is a constant value which satisfies 0.5 ≤ μ_A_(x_mn_) ≤ 1 condition, and μ_b_ and μ_o_ are the background and object mean, respectively.

For a given threshold value, the membership of a pixel is assigned to a class (object or background) by the absolute difference between the gray level and the average gray level of its belonging class. If this absolute value is high then the pixel membership value becomes smaller. The thresholding technique creates a binary image from where the area occupied by soil aggregate can be calculated.

**Figure 3:**
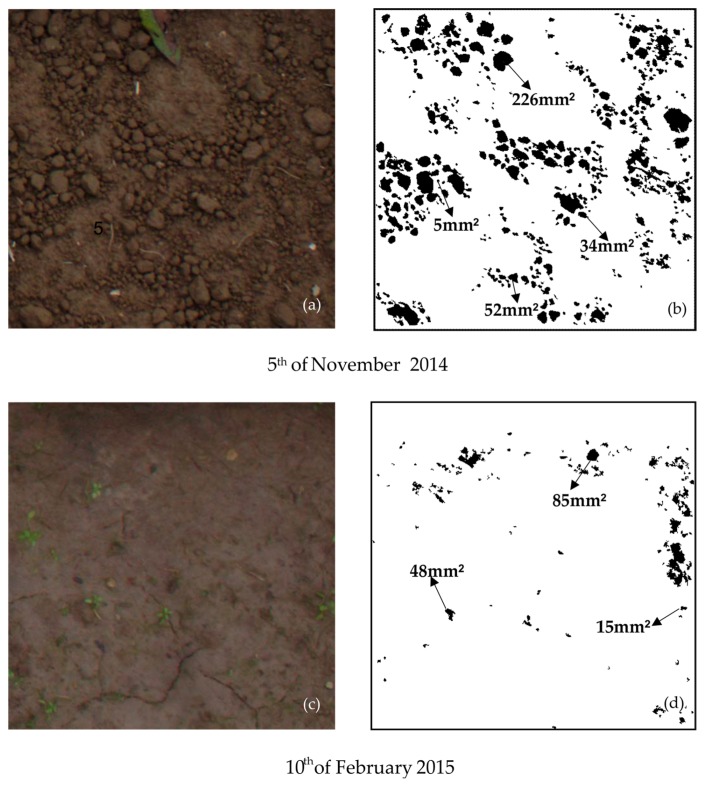
On the left side are original images (**a**,**c**) of Soil 1. Using the Huang thresholding technique, soil aggregates (black color) on the right side (**b**,**d**) are defined. While images (**a**,**b**) show the results at the beginning of the experiment (5 November), images (**c**,**d**) show the results at end of the experiment (10 February). The area of some aggregates calculated in mm^2^ is shown as an example.

The threshold value of the equalized reference image (120, 107, 104, 99 and 108 for Soil 1, Soil 2, Soil 3, Soil 4 and Soil 5, respectively) was used to the other images of the corresponding dataset. Before calculating soil aggregate area, a calibration procedure that converts an image to metric units was completed. While for Soil 1 and Soil 4, the biggest soil aggregate size was 400 mm^2^, for the other soils it was 500 mm^2^. Therefore, soil aggregate sizes ranging from 2 mm^2^ to 400 mm^2^ or from 2 mm^2^ to 500 mm^2^ depending on the dataset were included in the calculation. These setting were not the same because each dataset had different soil aggregate sizes. The soil aggregates that were touching the image edges were excluded. Figure 3 shows an example of the Huang thresholding technique together with soil aggregates calculated in ImageJ 1.51c.

### 2.6. Weather Data Collection

The DAVIS weather station recorded data most of the time, but from 15 to 23 November 2014 we had missing data. The weather station at Twenthe airport, located 4 km away from our experimental site, was used to fill data gaps in the temperature recording. The two stations are mounted at different height. While DAVIS sensor is installed at 2 m, the sensor at Twenthe airport is at 1.5 m above ground. In addition, the air temperature sensor has a nominal accuracy of ±0.5 °C and ±0.1 °C for DAVIS and Twenthe station, respectively. Despite these differences, we found a correlation with a coefficient of determination R^2^ = 0.96 for the minimum air temperature between the two stations. For daily rainfall data, the coefficient of determination between the two stations was R^2^ = 0.31, meaning that this data could not be used. The weather data of Twenthe airport were obtained from the website of the Royal Netherlands Meteorological Institute [54].

## 3. Results

**Figure 4:**
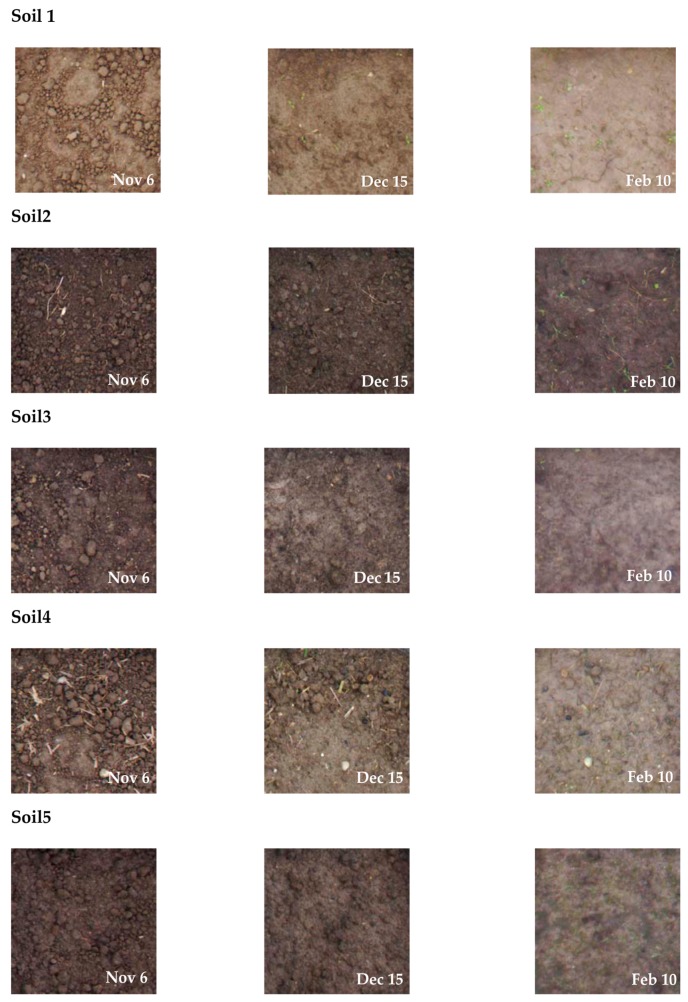
Original images for all soils at the beginning of the experiment (6 November 2014 images), after the first cycle of freezing–thawing followed by the biggest rain event (15 December 2014 images) and at the end of the experiment (10 February 2015 images). All soils experienced aggregate breakdown over time.

Figure 4 shows the original images at three moments in time at the beginning of the experiment (on 6 November 2014), after the first cycle of freezing–thawing followed by biggest rain events (on 15 December 2014), and at the end of the experiment (on 10 February 2015). These observations showed changes at the soil surface over time.

### 3.1. Weather Data

For the period that the experiment ran, most of the time rainfall intensity was mostly less than 2.5 mm·h^−1^ (light rainfall). However, on 12 and 19 December 2014 and on 2, 8, 10, 13, 15, and 28 January 2015, the rainfall intensity was more than 2.5 mm h^−1^. We considered here daily rainfall data because the images came at the daily base. Since we ran the experiment at winter time, freezing intervals occurred on 1–4 December 2014, 26–30 December 2014, 20–24 January 2015, and 3–7 February 2015. These four freezing–thawing cycles were followed by rain events on 7–24 December 2014, 2–15 January 2015, 25–31 January 2015 and 9–10 February 2015, respectively. The minimum air temperature recorded at the Twenthe weather station was used to fill the data gap.

### 3.2. Shadow Ratio

**Figure 5:**
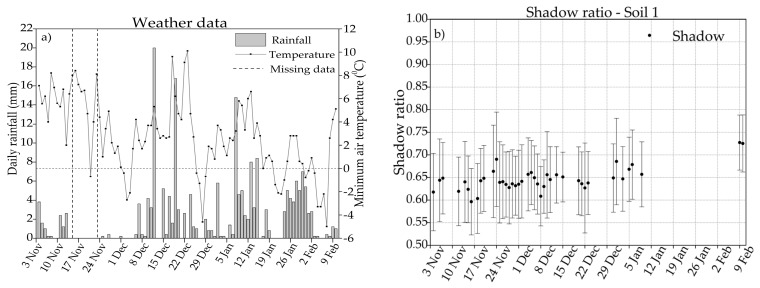

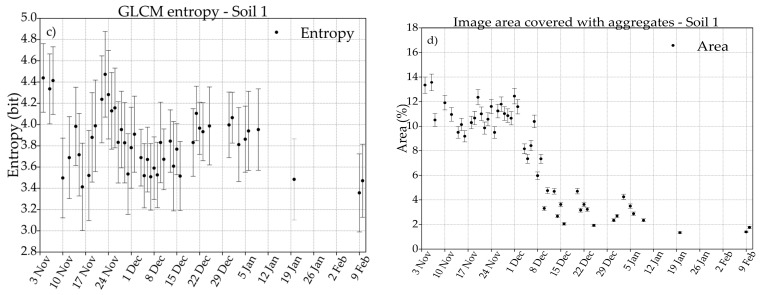
Weather data (**a**) together with shadow ratio (**b**); entropy (**c**); and area (**d**) results of Soil 1 are shown. Gray bars and gray line indicate daily rainfall and minimum air temperature, respectively. The gray dashed horizontal line indicates the temperature in °C. The vertical black dashed lines show missing rainfall data interval from 15 to 23 November 2014. The error bars indicate the standard deviation of: shadow ratio (**b**); and entropy (**c**). The standard error bars of area (**d**) represent the 95% confidence interval of the true population mean for the sample size 49.

**Figure 6:**
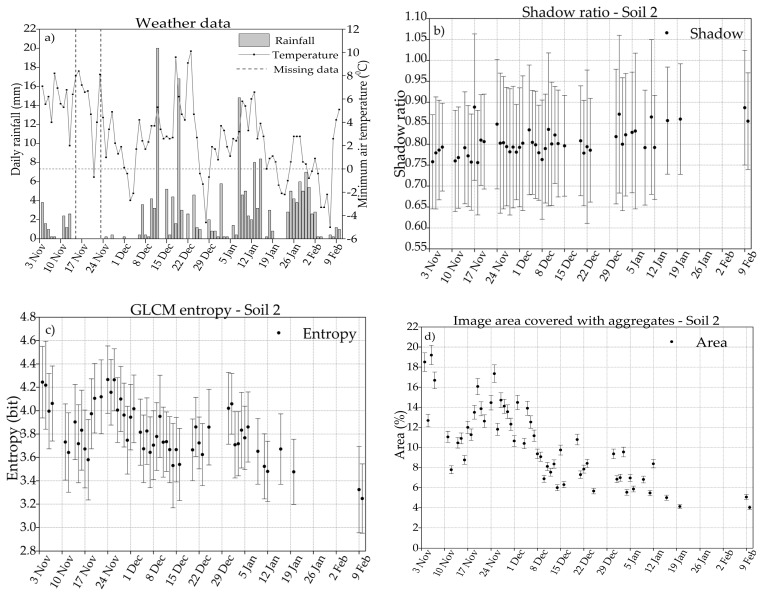
Weather data (**a**) together with shadow ratio (**b**); entropy (**c**); and area (**d**) results of Soil 2 are shown. Gray bars and gray line indicate daily rainfall and minimum air temperature, respectively. The gray dashed horizontal line indicates the temperature in °C. The vertical black dashed lines show missing rainfall data interval from 15 to 23 November 2014. The error bars indicate the standard deviation of: shadow ratio (**b**); and entropy (**c**). The standard error bars of area (**d**) represent the 95% confidence interval of the true population mean for the sample size 56.

**Figure 7:**
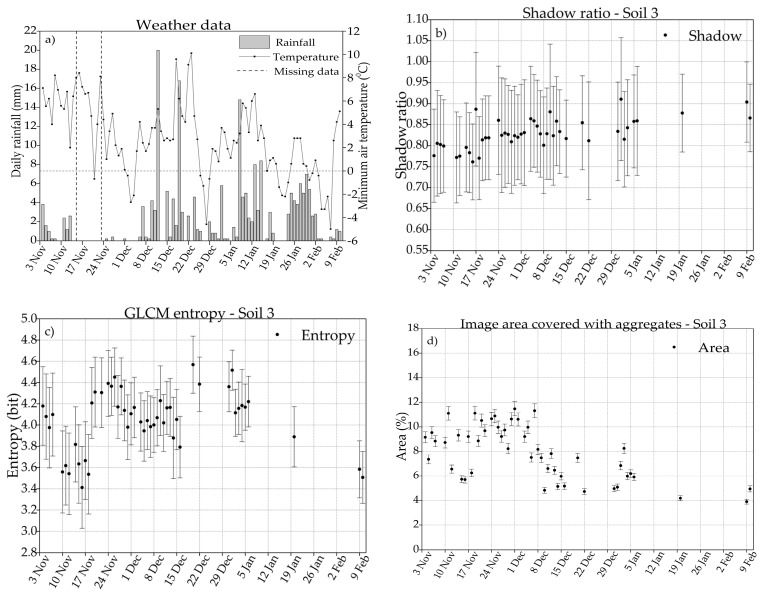
Weather data (**a**) together with shadow ratio (**b**); entropy (**c**); and area (**d**) results of Soil 3 are shown. Gray bars and gray line indicate daily rainfall and minimum air temperature, respectively. The gray dashed horizontal line indicates the temperature in °C. The vertical black dashed lines show missing rainfall data interval from 15 to 23 November 2014. The error bars indicate the standard deviation of: shadow ratio (**b**); and entropy (**c**). The standard error bars of area (**d**) represent the 95% confidence interval of the true population mean for the sample size 50.

**Figure 8:**
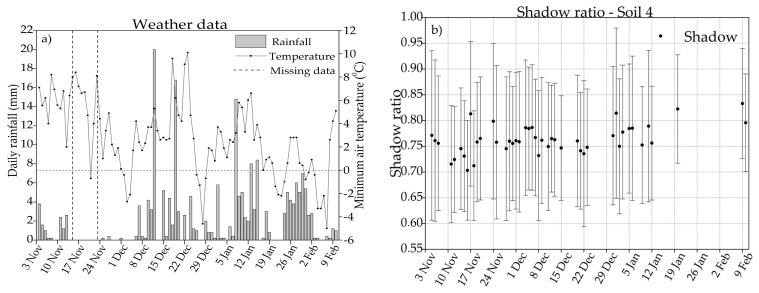

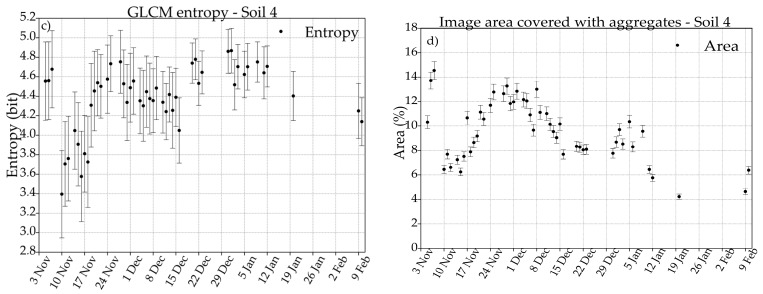
Weather data (**a**) together with shadow ratio (**b**), entropy (**c**); and area (**d**) results of Soil 4 are shown. Gray bars and gray line indicate daily rainfall and minimum air temperature, respectively. The gray dashed horizontal line indicates the temperature in °C. The vertical black dashed lines show missing rainfall data interval from 15 to 23 November 2014. The error bars indicate the standard deviation of: shadow ratio (**b**); and entropy (**c**). The standard error bars of area (**d**) represent the 95% confidence interval of the true population mean for the sample size 50.

**Figure 9:**
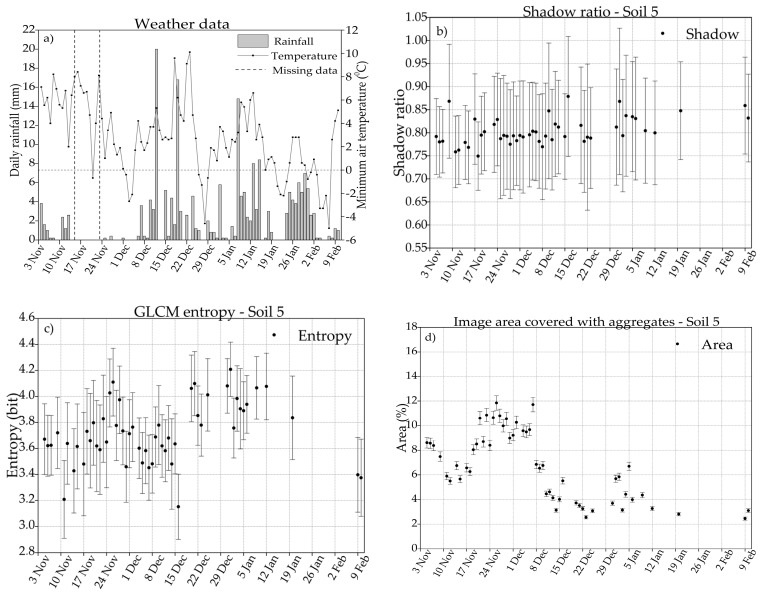
Weather data (**a**) together with shadow ratio (**b**), entropy (**c**); and area (**d**) results of Soil 5 are shown. Gray bars and gray line indicate daily rainfall and minimum air temperature, respectively. The gray dashed horizontal line indicates the temperature in °C. The vertical black dashed lines show missing rainfall data interval from 15 to 23 November 2014. The error bars indicate the standard deviation of: shadow ratio (**b**); and entropy (**c**). The standard error bars of area (**d**) represent the 95% confidence interval of the true population mean for the sample size 54.

The shadow ratio showed a similar trend for all the soil types (Figure 5b, Figure 6b, Figure 7b, Figure 8b and Figure 9b). Weather conditions (freezing–thawing cycles and rain events) seem to have no effect on the shadow ratio. The biggest rain event (20 mm day^−1^) on 12 December does not decrease the shadow ratio, although soil splash material was observed at the side of soil trays. In February, the shadow ratio is higher compared to November. Below the results of shadow ratio at the start and at the end of the experiment together with standard deviation were shown. The shadow ratio of Soil 1 varied from 0.62 (±0.09) to 0.73 (±0.06). Only in January and afterword’s the shadow had the tendency to increase (Figure 5b). Soil 1 (Silty Loam with 4.6% OM) is subject to crust development associated with cracks on the soil surface when exposed to various stresses (Figure 4). The shadow ratio of Soil 2 varies from 0.76 (±0.11) to 0.85 (±0.12). Apart from some fluctuations in shadow ratio, Soil 2 shows the same behavior as Soil 1 (Figure 6b). The shadow ratio of Soil 3 varies from 0.78 (±0.11) to 0.87 (±0.08). The shadow ratio stays more or less constant during November and December. An increasing trend is observed in January (Figure 7b). The shadow ratio of Soil 4 (Figure 8b) varies from 0.77 (±0.16) to 0.80 (±0.10). This soil has stones on its composition, which are more noticeable at the end of the experiment. The trend of shadow ratio of Soil 4 is similar with Soil 2. Shadow ratio of Soil 5 (Figure 9b) varies from 0.79 (±0.08) to 0.83 (±0.10) and it has a similar trend with the shadow ratio of Soil 1. The soil surface smooths out over time causing high soil reflectance value. If a smooth surface would have been reached, the value of the shadow ratio would have become constant. When our experiment ended, the process of aggregate breakdown was still ongoing, and therefore the ratio was still increasing.

### 3.3. GLCM Entropy

In Figure 5c, Figure 6c, Figure 7c, Figure 8c and Figure 9c, it can be seen that the GLCM entropy changes over time for all the soil types following both freezing–thawing cycles and rain events (Section 3.1). Due to light rainfall the entropy decreases. The entropy decreases further with the rain event of 12 December 2014 with a total rainfall of 20 mm day^−1^. While this behavior is notable in November and December (Figure 5c, Figure 6c, Figure 7c, Figure 8c and Figure 9c), it is less obvious in January and February because of missing image data for these months. When there is no rain or the rainfall amount is 0.2 mm day^−1^ the entropy increases. During the freezing period (1–4 December 2014) an increase in entropy is noticed. As soon as the thawing period starts (on 5 December 2014) the entropy decreases. Below the results at the start and at the end of the experiment together with the standard deviation are shown. Entropy of Soil 1 varies from 4.44 (±0.32) to 3.47 (±0.34) bits. A decrease of entropy is observed after the biggest rain event (20 mm day^−1^) occurred. Moreover, after two cycles of freezing–thawing followed by rain events (beginning and the end of December) the entropy has tendency to decrease (Figure 5c). The entropy of Soil 2 varies from 4.24 (±0.31) to 3.25 (±0.30) bits. Here as well the freezing followed by thawing increases and decreases the entropy, respectively. As a result of the big rain event (on 12 December 2014), the entropy decreases. In January the entropy shows a decreasing trend (Figure 6c). The entropy of Soil 3 varies from 4.18 (±0.37) to 3.51 (±0.24) bits. Although the entropy decreases when a rain event occurs and it increases during dry days the changes are small. The freezing–thawing cycles followed by the rain events (beginning and end of December) do not affect much the entropy. However, after 15 January 2015 entropy decrease is obvious (Figure 7c). The entropy of Soil 4 varies from 4.56 (±0.40) to 4.14 (±0.25) bits. This soil shows first a decrease in entropy. In addition, the biggest rain event on 12 December 2014 causes a decrease in entropy. Due to freezing the entropy increases. However, it does not decrease during thawing period (after 5 December). For the rest of experiment the entropy stays high showing a small decreasing trend (Figure 8c). The entropy of Soil 5 varies from 3.67 (±0.27) to 3.37 (±0.30) bits. The entropy follows the freezing–thawing cycles and rain events like the other soils. However, the entropy fluctuations are smaller compared to other soils. From 12 January 2015 the entropy decreases continuously (Figure 9c).

### 3.4. Huang Thresholding Technique

The image area covered with soil aggregates decreases over time (Figure 5d, Figure 6d, Figure 7d, Figure 8d and Figure 9d). The percentage of area does not change much during November. The trigger that initiates the decrease of area covered with aggregates is freezing–thawing followed by the rain events in December. This is notable for all the soils. This is the moment that the percentage of area covered with soil aggregates reduces significantly. Below the results at the start and at the end of the experiment together with the standard error (SE) are shown. For Soil 1 the image area covered with soil aggregates decreased from 13.57% (±0.66) to 1.76% (±0.09). In November the area covered with soil aggregates remains more or less constant. After the first freezing event (1–4 December 2014) the decrease of area covered with aggregates is evident (Figure 5d). Soil 2 trend is similar to Soil 1. Image area covered with soil aggregates decreased from 19.2% (±0.96) to 4.03% (±0.20). The freezing event (1–4 December 2014) causes a decrease in area percentage. After the biggest rain event (20 mm day^−1^) on 12 December the changes are small (Figure 6d). Image area of Soil 3 covered with aggregates decreased from 11.47% (±0.57) to 3.9% (±0.20). The area decreases because of the first freezing–thawing event (1–4 December 2014). After this moment the freezing–thawing followed by rain events had little influence on the area (Figure 7d). The area covered with soil aggregates of Soil 4 decreased from 14.54% (±0.73) to 4.23% (±0.21). This soil shows a different trend compared to previous soils. However, like the other soils the first freezing–thawing (1–4 December 2014) decreased the area (Figure 8d). For Soil 5 the image area decreased from 11.85% (±0.59) to 2.45% (±0.12). Soil 5 has a similar trend with Soil 4 in November. The first freezing–thawing (1–4 December 2014) and the rain events on 12 and 19 December 2014 decreased the area covered with aggregate (Figure 9d). After the first freezing event the SE of all the soils gets smaller. This suggests that the number of soil aggregates decrease due to freezing–thawing cycle.

**Figure 10:**
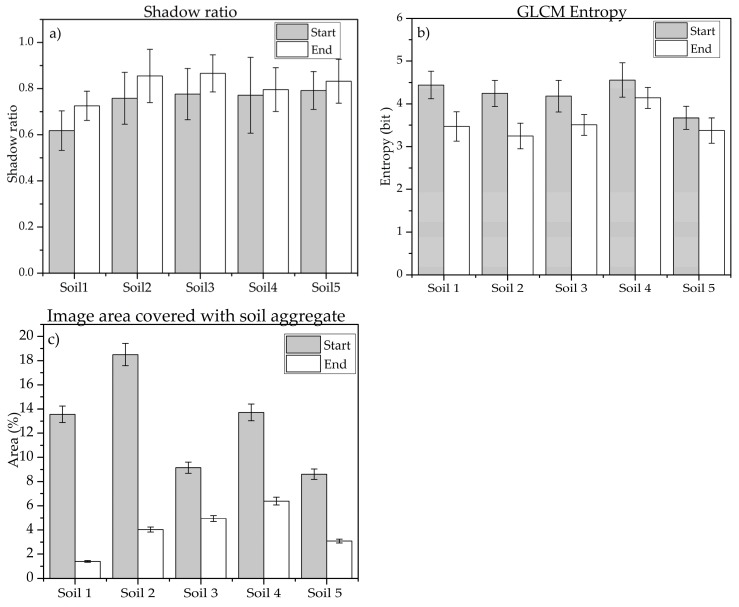
Summary results of shadow ratio, entropy and area covered with aggregates obtained for all soils using: band ratio (blue/red) (**a**); GLCM entropy (**b**); and Huang thresholding (**c**) approaches. The grey and white bars indicate the start and the end of the experiment, respectively. Each dataset is relative to each tray and cannot be taken as an absolute value.

Figure 10 summarizes the results of shadow ratio, entropy, and image area covered with soil aggregates at the start and at the end of experiment for all the soils. We observe that the shadow ratio is higher at the end of the experiment (Figure 10a). Both the GLCM entropy and the image area cover with soil aggregates decrease at the end of experiment, as observed in Figure 10b,c, respectively.

## 4. Discussion

We hypothesize that over time soil surface becomes smooth, because rainfall or freezing–thawing cycles destroys soil aggregate (Figure 4).

### 4.1. Shadow Ratio

For all the soil types the shadow ratio shows a gradual change over time. Although the soil aggregates break down because of freezing–thawing cycles followed by rain events, the shadow ratio does not show details related to weather conditions. In February, the shadow ratio is higher compared to November. These results are related with high reflectance of a smooth soil surface at the end of the experiment. When both bands (blue/red) have the same reflectance value the shadow ratio becomes constant. The constant shadow ratio indicates that no changes occur at the soil surface. The high shadow ratio at the end of the experiment is more noticeable for Soil 1, Soil 2 and Soil 3 than for Soil 4 and Soil 5 (Figure 10a). Due to crust formation at the end of the experiment, the reflectance and consequently the shadow ratio of Soil 1 is high. This is what we expected for Soil 1. Similar to Soil 1, Soils 2 and 3 are Silty Loam, but they have high OM content. Soil aggregates are formed by bonding of clay particle, polyvalent minerals (Fe, Al, Ca) and organic matter [55]. Therefore, the organic matter is important not only for soil fertility, but also for soil structure stability. Although Soil 2 is from agricultural land and Soil 3 is from a forest area, the shadow ratio of these soils is similar at the end of the experiment. This could be related with a surface crust formation especially for Soil 3. These soils are subject to frequent crust development. Therefore, the creation of the white layer could be a result of this process. Moreover, appearance of wild plants in Soil 2 is noticeable (Figure 4). At the end of the experiment the shadow ratio of Soil 4 (Loam) and Soil 5 (Sandy Loam) increases 0.03 and 0.04, respectively, when compared to the start of the experiment. The low increase of shadow ratio at the end of the experiment could be related with BRDF, for which we did not correct. With a 60° horizontal field of view, the camera observed more shadow in aggregates viewed towards the sun (Soils 1 and 2) than in aggregates viewed away from the sun (Soils 4 and 5). This implies that interpreted results can be compared between the different trays, but that data values cannot be compared between trays in absolute terms. The difference in solar azimuth over time is 6.7°, which is negligible for the three measures presented in this paper. The difference in solar elevation can however lead to a ~50% difference in shadow length. Different from solar azimuth, we could expect that changing solar elevation would be visible in the shadow ratio, but the results do not show that at all. A longer time series might have brought this to light. Considering the mean as a signal and the standard deviation as a noise, the Signal-to-Noise ratio (SNR) for each band could be calculated. The high SNR value indicates that the band has more information than noise. For Soil 1 the SNR of red band was higher compared to SNR of blue band. The opposite was true for other soils. This is the reason why shadow ratio (blue/red) of Soil 1 was lower compared to other soils. Moreover, the standard deviation of shadow ratio is relatively large, because it is related to the number of pixels in the image (512 × 512 pixels).

### 4.2. GLCM Entropy

The entropy was able to follow changes on soil surface for all the soils used in this study. The high and low entropy value indicates that image is heterogeneous and texturally uniform, respectively. Figure 10b shows that the entropy is higher at the beginning of the experiment compared to the end of the experiment. Although rainfall intensity was low (<2.5 mm h^−1^) most of the time that the experiment ran, the soils were at a wet condition. In fact, soil water content at time of freezing is important because, as the air temperature drops below 0 °C, the soil temperature gets low causing frozen soil. As a result, the ice crystals expand in pores between soil particles affecting particle-to-particle bond [56]. During freezing days, the entropy was high because the frozen soils had many ways to arrange themselves, revealing a dispersed distribution of soil aggregates. As soon as the thawing period starts the soil particles tend to bond again. At this time the entropy stayed constant or decreased, because soil aggregates had a limited number of ways to bond together indicating a compact distribution with few grey level variations. The GLCM entropy showed the same trend for all the soils except the magnitude which differed with soil type. This is of course related to soil properties affecting soil aggregate breakdown. Clay is one of the aggregating factors in a soil [57,58]. However, the effect is different depending on its mineralogy. X-ray diffraction soil samples analysis indicated that the dominant clay mineral was kaolinite. In addition, organic matter is responsible for soil structure stability [59,60]. Both, Soil 1 and Soil 4 have low OM and high percentage of silt particles making these soils vulnerable to freezing–thawing cycles and to rain events. However, the entropy changes for Soil 4 are smaller compared to Soil 1 over time. This could be related not only with the stony composition of Soil 4, but also with a lower silt amount (44%) in Soil 4 compared to Soil 1 (71%). Soil 2 (agricultural land) had lower entropy value than Soil 3 (forested land) at the end of the experiment. This means that land use/land cover has an effect on soil resistance to freezing–thawing and rainfall impact. Unlike shadow ratio, GLCM entropy was able to distinguish the importance of land use/land cover on soil aggregate breakdown. The entropy of Sandy Loam was calculated over a more homogeneous image (similar aggregate size) with less intensity variations compared to other soils. This is a reason why Sandy Loam had lower entropy values. This soil type drains and dries out more rapidly than the clayey soils. Therefore, freezing has little effect on dried soil indicating the importance of soil water content at freezing time. Entropy is not calculated at the edges of an image. Although the image size decreases depending on the window size used, the standard deviation of entropy is relatively large because it depends on the number of analysis windows.

### 4.3. The Huang Thresholding Technique

The results obtained using the Huang thresholding technique were the best (short error bars indicate high precision) compared to both shadow ratio and GLCM entropy (wide error bars indicate large error). The Huang thresholding method was able to quantify the soil aggregates changes over time. As mentioned in Section 4.1 and Section 4.2 (shadow ratio and GLCM entropy), it is the combination of freezing–thawing cycles followed by rain events that triggers soil aggregate breakdown.

Soil aggregate stability is its ability to maintain its structural arrangement and void spaces when exposed to various stresses. Freezing–thawing cycles affect soil aggregate stability [61,62]. However, this effect varies with soil texture, OM content, initial aggregate size, soil water content at the time of freezing, freezing temperature, number of freezing–thawing cycles. Likewise, raindrop impact affects the soil surface in different ways. When a soil is dry, aggregate disruption by splashing is most effective [63,64]. The impact of raindrop on a wet soil plays a role in compaction [31]. In addition, Algayer et al. [65] showed that soil aggregate stability varied in few days and this was related with soil water content and rainfall intensity.

It is interesting to observe that Soil 2 with high OM content had the highest loss of soil aggregates compared to other soils as it is shown in Figure 10c. Soil 2 is from an agricultural land. The high OM content in Soil 2 is probably coming from sewage sludge manure mixed with plant residues application by the farmer some days before soil sample was taken. The manure had probably not yet mixed properly with the soil matrix since it is not normal to have such a high amount of OM in agricultural fields. Soil 3 with high OM is from forested area. This soil had the lowest soil aggregates loss compared to other soils. In a study of accumulated effect of rain on micro-topographic erosion features in various land uses under natural conditions [66] reported higher erosion hazard in agricultural fields and lower in forest cover indicating the pronounced effect of land cover on aggregate stability. Our results also indicate that forested soils are more resistant to freezing–thawing cycles followed by rain events than agricultural ones. Although the OM content of Soil 2 and 3 is very similar, they act differently under freezing and thawing. This could be related to the difference in origin in OM. Soil 2 sewage sludge could have higher water retention characteristics than the more apolar OM of forested soils (Soil 3). Therefore, Soil 2 (agricultural soil) expansion during freezing result in more aggregate breakdown compare to Soil 3 (forested soil).

As mentioned in Section 4.2 (GLCM entropy), this soil drains fast and freezing has little influence on it. Soil 1 (Silty Loam) and Soil 4 (Loam) both have low OM, but different amount of silt particles which make them behave differently. In fact, Soil 1 at the end of the experiment shows crust formation which cannot be seen for Soil 4 (Figure 4). Besides the soil properties, these results are influenced by soil aggregate size at beginning of the experiment. In Section 2.5 we specified that the smallest aggregate size is 2 mm^2^ and the biggest one is 400 mm^2^ (Soil 1 and Soil 4) and 500 mm^2^ for the other soils. Within this range (2–400 or 2–500 mm^2^), soil aggregate sizes vary significantly. Although we were not able to follow each soil aggregate change over time, at the end of the experiment the average soil aggregate size was 16 mm^2^, 12 mm^2^, 17 mm^2^, 17 mm^2^, 13 mm^2^ for Soil 1, Soil 2, Soil 3, Soil 4 and Soil 5, respectively. Moreover, by visual inspection, we observed that the size of aggregates of Soil 2 is smaller compared to other soils at the beginning of the experiment (Figure 4). These results might indicate that while small aggregates are destroyed the big ones decrease in size. This is the objective of the following work to quantify how much individual soil aggregate change over time.

When looking at relative changes over time, no absolute calibration per se is needed. Both the band ratio and the gray level matrix do not depend on absolute data values, while the Huang technique was performed on data normalized with histogram matching on each of the 5 image subsets separately. If, for example, vegetation would have started to grow, a histogram matching normalization would likely not suffice. In our experiment, however, there was only bare soil during the experiment. There was no apparent change in surface composition, other than changes in sand and clay fractions.

In soil erosion models it is well recognized the importance of soil surface conditions and the need to take into account their spatial and temporal heterogeneity. RS can play a significant role in acquiring relevant data for soil erosion models in order to improve their performance.

Besides climate, in an agricultural area soil aggregates are affected by cultivation practices. Soil is less exposed to raindrop impact during the cropping season due to crop canopy. It is important to identify periods of low stability when the soil is particularly vulnerable to structural breakdown. Good ground cover is an important part of the majority of soil conservation programs. At this stage of our research, the potential users can be the scientific community dealing with soil erosion modeling and environmental concern. Further investigation is needed to make these results practical to the decision-makers and farmers.

## 5. Conclusions

In this study, band ratio, image texture analysis and object based image analysis derived from normal photography were used to monitor soil aggregate break down under natural conditions. Five different soils were chosen, varying in texture classes (Silty Loam, Sandy Loam and Loam) and organic matter content (agricultural soil and forest soil). Trays with aggregates were exposed to outside weather circumstances during winter season, with exposure to rainfall and freezing–thawing. The winter season (October to March) was used as the soils in the Netherlands are normally bare and aggregate breakdown takes place. Images with fog, inundation, freezing and inhomogeneous lighting were discarded. A weather station adjacent to the plots recorded all meteorological variables.

The assumption is that the aggregates break down under the kinetic energy of rainfall and freezing–thawing, and smooth the surface. A decrease in aggregate size and loss of aggregates should be visible in the image series, and different indices derived from these images might serve as roughness indicators. Of the three image derived indices, shadow ratio is the worst. The assumption is that large aggregates cast a larger shadow than small aggregates, but in reality there are many roughness elements that cause shadows, also because the sun during winter is at a low angle (13.6–17.5° at our experimental site). The effect of aggregate breakdown cannot be well observed and there is no relation between shadow ratio and rainfall or air temperature. The texture index (GLCM entropy) is moderately successful in the decreasing trend with cumulative rainfall but the index is complex to compute and has to be optimized for a given situation. Both shadow ratio and GLCM entropy have relatively large standard deviations. The Huang thresholding technique captures the aggregates themselves and successfully shows a decrease in size, with a low standard deviation. Given sufficient resolution of the images, aggregate breakdown can be followed by this method. The low standard deviation is logical as the number of aggregates is much smaller than the number of pixels (shadow ratio) or number of analysis windows (GLCM entropy). Therefore, standard deviation should not be used as a comparative among approaches.

## Figures and Tables

**Table 1:** The soils used in this study. Soils 1–4 (Silty Loam and Loam) were sampled in the Limburg province, the Netherlands, and Soil 5 (Sandy Loam) was sampled in the city of Deventer, the Netherlands. The agricultural crop cultivated on all the fields was maize. However, at the time of soil sampling this crop was already harvested.

SoilID	Location (WGS 84)		Soil Particle Size (%)			Texture Class	OM (%)	Land Use
	Lat	Long	Clay	Silt	Sand			
Soil 1	50.7758°	5.8824°	16	71	13	Silty Loam	4.6	Agriculture
Soil 2	50.7687°	5.9201°	23	52	25	Silty Loam	12.3	Agriculture
Soil 3	50.7727°	5.9213°	22	54	24	Silty Loam	12.5	Forest
Soil 4	50.8694°	5.7884°	17	44	39	Loam	5.6	Agriculture
Soil 5	52.2810°	6.1813°	14	11	75	Sandy Loam	5.3	Agriculture
